# Vascularised fibular graft shoulder arthrodesis after proximal humeral tumour resection with sacrifice of the abductor mechanism

**DOI:** 10.1007/s00264-026-06934-9

**Published:** 2026-07-09

**Authors:** Silvia Brotea - Mosoiu, Ovidiu - Gogu Cacuci, Ruxandra Bodog, Sergiu Samuila, Florian Bodog, Adyb - Adrian Khal

**Affiliations:** 1 Department of Oncology, Carol Davila University of Medicine and Pharmacy, Bucharest, Romania; 2 Department of Oncology, Institute of Oncology Alexandru Trestioreanu, Bucharest, Romania; 3 Faculty of Medicine, University of Oradea, Oradea, Romania; 4 Department Of Orthopaedics, Pelican Medicover Hospital, Oradea, Romania; 5 Department of Plastic Surgery, Pelican Medicover Hospital, Oradea, Romania; 6 Department of Paediatric Orthopaedics, AP-HM Timone Enfants, Marseille, France

**Keywords:** Proximal humerus, Bone tumour, Arthrodesis, Vascularized fibula, Limb salvage, Scapulothoracic compensation

## Abstract

**Purpose:**

Reconstruction after proximal humeral tumour resection remains challenging when tumour clearance requires sacrifice of the shoulder abductor mechanism. This study evaluated oncological, radiological, functional, and cosmetic outcomes after shoulder arthrodesis using a vascularized fibular graft.

**Methods:**

We retrospectively reviewed 12 patients who underwent proximal humeral tumour resection and shoulder arthrodesis with a vascularized fibular graft between 2019 and 2025. All resections involved the rotator cuff and resulted in loss of the functional deltoid mechanism. Outcomes included oncological status, union, graft hypertrophy, complications, MSTS score, range of motion, functional tasks, cosmetic outcome, and donor-site morbidity.

**Results:**

Mean age was 21 years and mean follow-up among survivors was 54 months. Diagnoses were osteosarcoma in nine patients, chondrosarcoma in two, and giant cell tumour in one. Margins were wide in 11 patients and marginal in one. Local recurrence occurred in one patient and distant metastases in three. Primary union was achieved in seven patients. Five developed distal graft-host pseudarthrosis; two underwent revision, of whom one united. Final bone union was achieved in eight patients. Among living patients, mean graft hypertrophy was 34%, mean MSTS score was 25.5/30, active flexion was 80°, and active scapulothoracic abduction was 75%. Cosmetic dissatisfaction was reported by all living patients.

**Conclusion:**

Shoulder arthrodesis with a vascularized fibular graft can provide a stable, functionally useful limb after proximal humeral tumour resection involving the abductor mechanism, although pseudarthrosis and cosmetic dissatisfaction remain important limitations.

## Introduction

Limb-salvage surgery is now the preferred treatment for most primary malignant and aggressive tumours of the proximal humerus, provided that adequate oncological margins can be achieved. However, reconstruction after proximal humeral resection remains one of the most demanding problems in musculoskeletal oncology. The reconstructive challenge is therefore defined not only by the length of humeral resection, but also by whether the rotator cuff, deltoid, and axillary nerve can be preserved. Consequently, postoperative function depends heavily on the extent of soft tissue sacrifice required for tumour clearance.

A variety of reconstructive options have been described, including modular endoprostheses, osteoarticular allografts, allograft-prosthetic composites, reverse shoulder arthroplasty, clavicula-pro-humero reconstruction, cement spacers, and biological arthrodesis [1–5]. None has proven universally superior, and the optimal choice remains dependent on tumour extent, patient age, expected survival, residual soft tissues, and functional goals. Reverse shoulder arthroplasty has expanded the reconstructive options available when the rotator cuff cannot be preserved, but its success relies on a functioning deltoid and intact axillary nerve [6]. In more extensive resections involving the deltoid insertion or axillary nerve, prosthetic reconstruction becomes less predictable and may be limited by instability, loosening, or revision surgery [7–9].

When oncological resection requires removal of the rotator cuff and results in loss of the functional deltoid mechanism, the goal of reconstruction changes. In this setting, restoration of active glenohumeral motion is rarely realistic. The priority becomes preservation of a stable, painless limb that allows the patient to position the hand in space through scapulothoracic compensation. Shoulder arthrodesis remains a relevant limb-salvage strategy for this selected indication. Previous studies have suggested that arthrodesis may provide reliable function after proximal humeral sarcoma resection involving the abductor mechanism, particularly when prosthetic or composite reconstructions are unlikely to restore active shoulder function [4, 10].

A vascularized fibular graft offers biological advantages in this context. It provides living autologous bone, allows reconstruction of large segmental defects, and has potential for incorporation, hypertrophy, and remodelling under mechanical load. Nevertheless, the technique is demanding and may be associated with delayed union, pseudarthrosis, graft fracture, donor-site morbidity, and cosmetic dissatisfaction.

The aim of this multicentre retrospective study was to evaluate the oncological, radiological, functional, and cosmetic outcomes of shoulder arthrodesis using a vascularized fibular graft after proximal humeral tumour resection involving the abductor mechanism. We hypothesised that, in this selected indication, the procedure would provide a stable and functionally useful limb despite loss of glenohumeral motion and potential biological and cosmetic complications.

## Materials and methods

### Study design and patients

This was a retrospective multicentre study of patients who underwent shoulder arthrodesis using a vascularized fibular graft after oncological resection of a proximal humeral tumour. Patients were identified from the surgical databases of two institutions between 2019 and 2025.

Inclusion criteria were: a primary malignant or aggressive proximal humeral tumour treated by limb-salvage resection; resection involving the shoulder abductor mechanism, including the rotator cuff and deltoid; reconstruction with shoulder arthrodesis using a vascularized fibular graft; and available clinical and radiological follow-up.

Details of chemotherapy, surgical margins, local recurrence, distant metastasis, survival, and status at final follow-up were collected from the medical records.

The study was approved by local ethics committee and the requirement for individual informed consent was waived because of the retrospective design and use of anonymised data.

### Oncological treatment and surgical procedure

All patients were discussed in a multidisciplinary tumour board before surgery. Staging investigations and perioperative oncological treatment were performed according to institutional sarcoma protocols and adapted to tumour histology and grade.

In all patients, resection included the proximal humerus and rotator cuff, with resection of the deltoid insertion and the anterior and lateral portions of the deltoid in most cases. The posterior deltoid was preserved when oncologically feasible. Overall, these resections resulted in loss of the functional shoulder abductor mechanism.

**Fig. 1:**
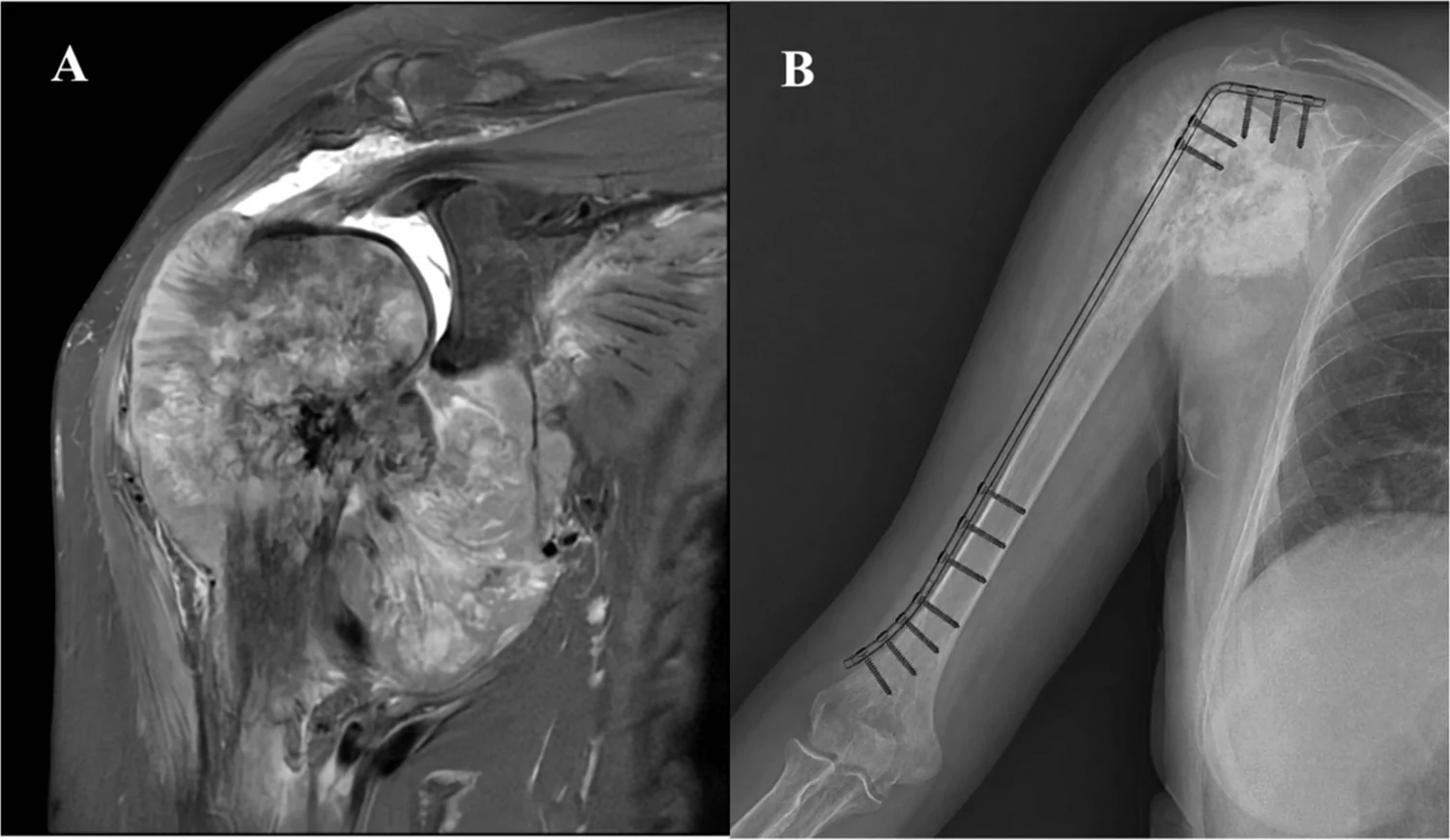
Preoperative planning for vascularized fibular graft shoulder arthrodesis. (**A**) Preoperative MRI demonstrating proximal humeral tumour extension and involvement of the shoulder abductor mechanism. (**B**) Preoperative planning radiograph showing the intended scapulohumeral arthrodesis construct, custom-made plate position, screw trajectory, and arthrodesis alignment

Reconstruction was performed during the same procedure using a free vascularized fibular graft harvested by the reconstructive microsurgical team. The graft was used to bridge the defect between the scapula/glenoid region and the remaining humeral shaft to achieve shoulder arthrodesis. Internal fixation was performed using a custom-made plate, with screw fixation alone used in selected cases. Arterial anastomosis was performed end-to-side between the donor peroneal artery and a major recipient artery at the host site. Venous anastomosis was performed end-to-end between the donor peroneal vein and a suitable adjacent recipient vein. The intended position of arthrodesis was 10° to 15° of flexion and abduction, with 35° to 45° of internal rotation (Fig. 1). Postoperatively, the upper limb was immobilised for three months, followed by progressive rehabilitation focused on elbow, wrist, hand, and scapulothoracic motion.

### Clinical and functional assessment

Patients were followed according to institutional oncological surveillance protocols. Clinical follow-up included assessment of pain, shoulder girdle stability, elbow and hand function, active upper-limb motion, complications, cosmetic appearance, and donor-site morbidity.

Functional assessment was performed in all living patients at final follow-up. Functional outcome was assessed using the Musculoskeletal Tumour Society (MSTS) score for the upper limb [11], reported as an absolute value out of 30 and as a percentage. Range-of-motion assessment included passive and active flexion, true shoulder abduction, scapulothoracic abduction, internal rotation and external rotation deficit. Validated patient-reported outcome measures, such as the DASH, QuickDASH, SF-36, or TESS, were not collected systematically during the study period and were therefore not included in the analysis.

Because the procedure aimed to achieve glenohumeral fusion, shoulder motion was analysed according to its functional origin. Active flexion was interpreted as total functional elevation generated mainly through scapulothoracic compensation and trunk motion rather than true glenohumeral motion. True shoulder abduction was recorded separately from scapulothoracic abduction.

Functional task outcomes were recorded when available, including the ability to bring the hand to the mouth, head, and pocket, and independence in basic activities of daily living.

Cosmetic outcome was assessed clinically and from patient-reported complaints. Donor-site morbidity was assessed by review of clinical notes and examination findings, including gait disturbance, ankle instability, pain, sensory symptoms, motor deficit, or deformity related to fibular graft harvest.

### Radiological assessment

Radiological follow-up consisted of serial plain radiographs of the shoulder and humerus. Radiographs were reviewed for graft position, fixation integrity, bone union, pseudarthrosis, graft fracture, implant failure, and fibular graft hypertrophy.

Union was assessed separately at the proximal arthrodesis site between the graft and the scapula/glenoid region and at the distal junction between the graft and the remaining humerus. Union was defined as bridging callus or continuous trabecular bone across the relevant junction on orthogonal serial radiographs, without persistent local pain, progressive radiolucency, or progressive implant failure. Time to union was recorded in months. Pseudarthrosis was defined as the absence of radiological union at final follow-up or the need for revision surgery for non-union. CT was not performed systematically and was therefore not used as a uniform radiological assessment method.

Fibular graft hypertrophy was measured on serial radiographs and expressed as a percentage increase compared with the immediate postoperative graft diameter according to the De Boer–El-Gammal formula [12, 13].

### Complications and oncological outcomes

Complications were recorded throughout follow-up, with particular attention to pseudarthrosis, graft fracture, fixation failure, infection, revision surgery, donor-site morbidity, cosmetic dissatisfaction, and oncological events. Donor-site morbidity included ankle instability, gait disturbance, neurological symptoms, pain, or deformity after fibular graft harvest.

Oncological outcomes included surgical margins, local recurrence, distant metastasis, death from disease, and status at final follow-up. Follow-up was calculated from the date of reconstruction to the most recent clinical review or death.

### Statistical analysis

Given the small sample size and rarity of the indication, analysis was descriptive. Continuous variables were reported as means and ranges. Categorical variables were reported as numbers and percentages. No comparative statistical testing was performed because there was no control group.

## Results

### Patient and tumour characteristics

We identified 12 patients. The mean age at surgery was 21 years (range 9 to 48). Histological diagnoses were osteosarcoma in nine patients, chondrosarcoma in two, and giant cell tumour in one. No patient had metastatic disease at diagnosis, and no patient received radiotherapy. Among surviving patients, the mean follow-up was 54 months (range 19 to 82).

### Surgical data

The rotator cuff was resected in all patients. The deltoid insertion and anterior/lateral deltoid were resected in most cases, with preservation of the posterior deltoid when oncologically feasible. The mean humeral resection length was 14.9 cm, and the mean vascularized fibular graft length was 16.3 cm.

### Oncological outcomes

All nine patients with osteosarcoma received neoadjuvant and adjuvant chemotherapy. Surgical margins were wide in 11 patients and marginal in one patient. Local recurrence occurred in one patient at eight months after surgery and was associated with metastatic progression. Distant metastases developed in three patients after a mean of 10.3 months (range 8 to 11). At final follow-up, nine patients were alive with no evidence of disease and three had died of disease at nine, 11, and 14 months postoperatively.

### Radiological outcomes

**Fig. 2:**
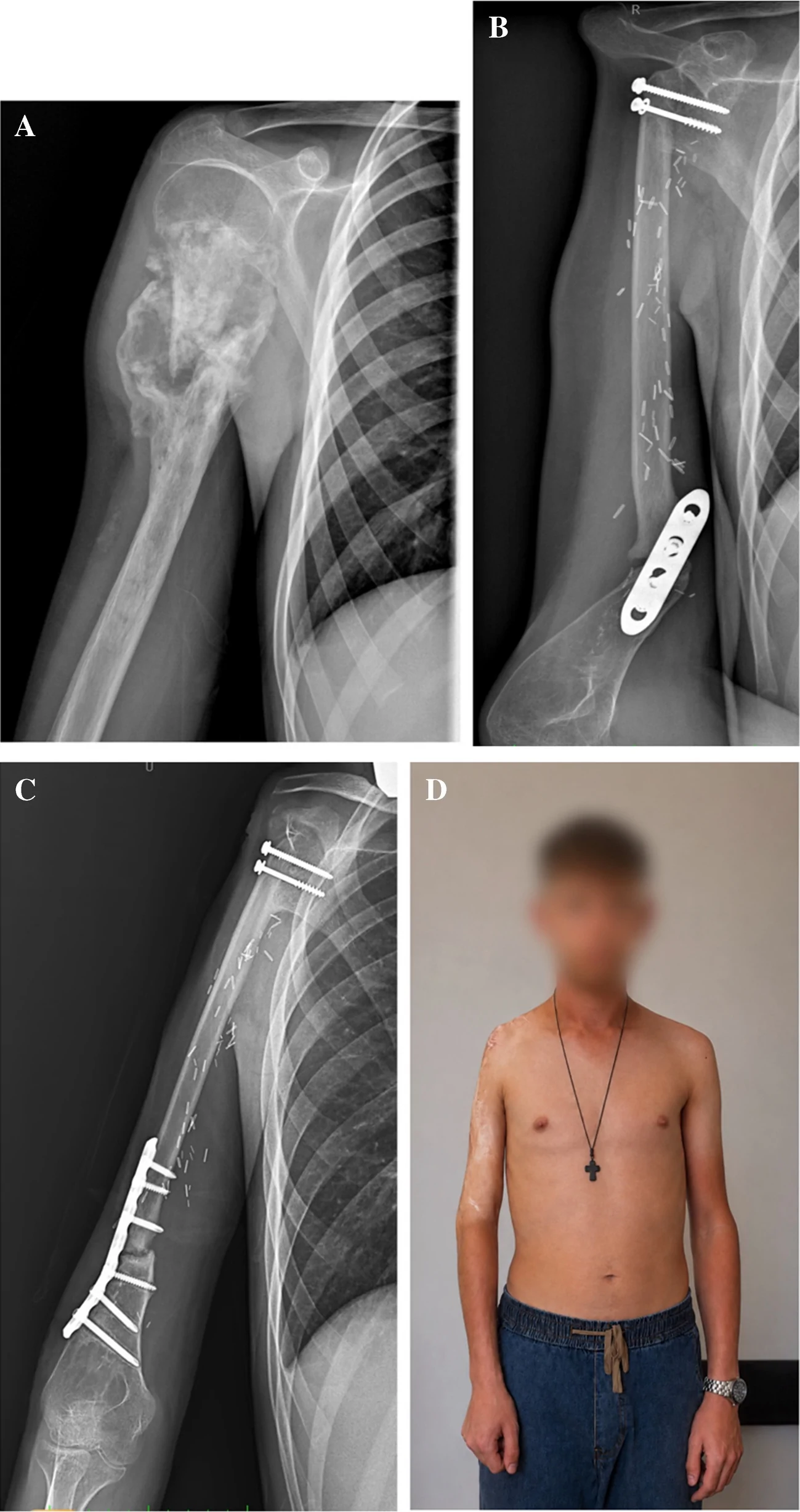
Persistent distal graft-host pseudarthrosis and cosmetic outcome after vascularized fibular graft shoulder arthrodesis. (**A**) Preoperative radiograph of a proximal humeral tumour. (**B**) Proximal union at the glenoid-side arthrodesis site, distal graft-host pseudarthrosis, and displacement of the distal plate after screw-only proximal fixation at 12 months postoperative. (**C**) Persistent distal graft-host pseudarthrosis with maintained proximal union at 3 years after the surgery. (**D**) Cosmetic asymmetry related to loss of shoulder contour and prominence of the reconstructed shoulder girdle

Primary bone union was achieved in seven of 12 patients (58%). Among patients who achieved union, the mean time to union was 23 months (range 4 to 68). Five patients developed pseudarthrosis, all at the distal graft-host junction between the vascularized fibular graft and the remaining humeral shaft (Fig. 2). No pseudarthrosis occurred at the proximal arthrodesis site. Two patients underwent revision surgery, of whom one subsequently united, while four had persistent non-union at final follow-up. Final bone union was therefore achieved in eight of 12 patients (66.7%). Among living patients, mean fibular graft hypertrophy was 34% (range 25% to 35%). In the three patients who died of disease, graft hypertrophy before death was 8%, 10%, and 23%, respectively.

### Functional outcomes

**Fig. 3:**
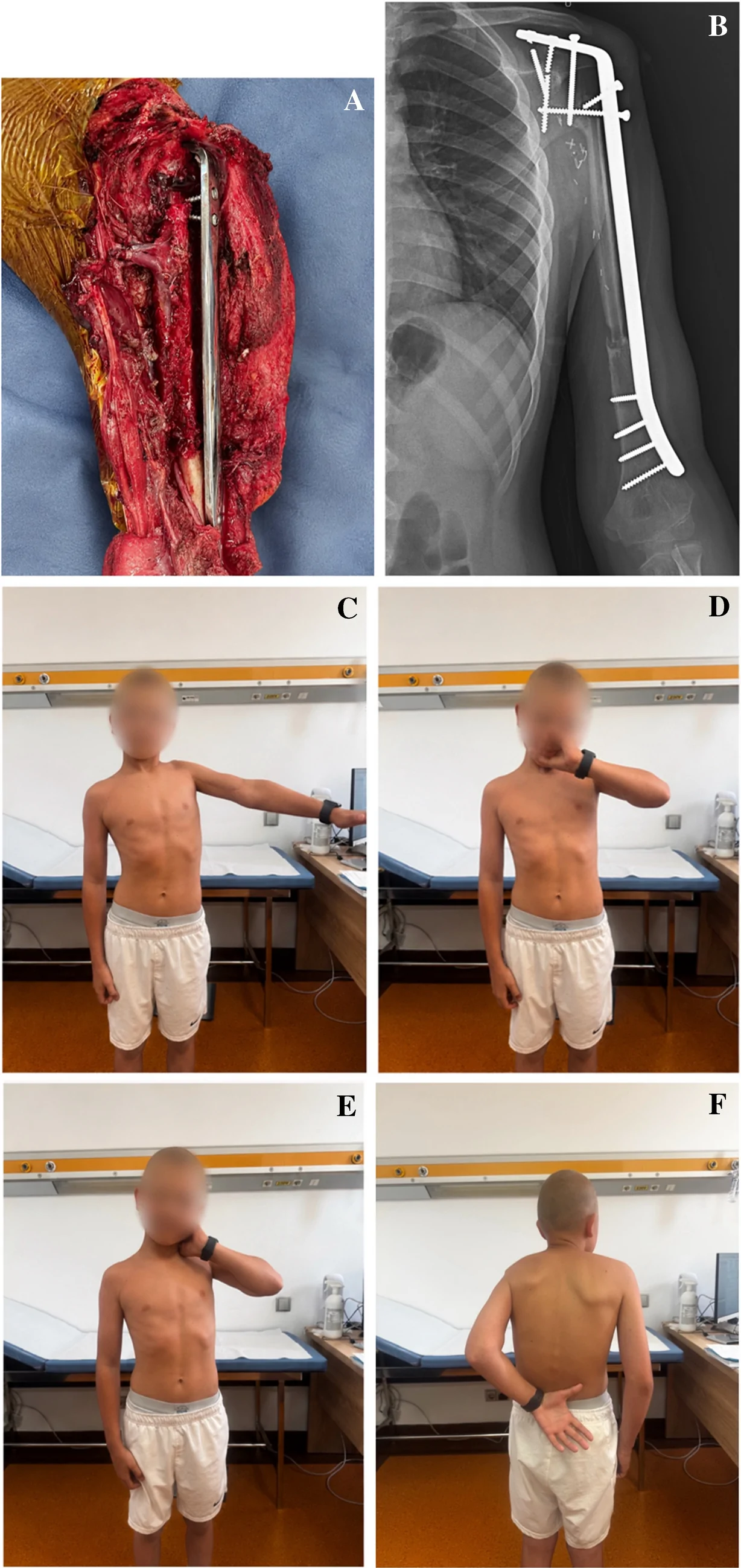
Vascularized fibular graft shoulder arthrodesis after proximal humeral osteosarcoma resection in a 10-year-old boy. (**A**) Intraoperative view of the vascularized fibular graft, plate fixation, and vascular anastomosis. (**B**) Proximal union at the glenoid-side arthrodesis site, distal graft-host pseudarthrosis, and fracture of two proximal screws at eight-month postoperatively. (**C–F**) Preserved functional limb positioning despite distal pseudarthrosis: abduction/elevation, hand-to-mouth, hand-to-head, and hand-to-back movements at eight-months after the surgery

Functional assessment was available for all living patients at final follow-up. Among the nine surviving patients at final follow-up, the mean MSTS score was 25.5 out of 30, corresponding to 85%. Mean passive flexion was 88° (range 85° to 100°), and mean active flexion, representing total functional elevation, was 80° (range 75° to 90°) (Fig. 3). Mean true shoulder abduction was 7° (range 0° to 20°). Functional abduction was achieved mainly through scapulothoracic compensation, with mean passive scapulothoracic abduction of 86° (range 70° to 110°) and mean active scapulothoracic abduction of 75° (range 70° to 80°). No patient reported scapulothoracic pain. Mean external rotation deficit was 27° passively (range 10° to 45°) and 32° actively (range 10° to 55°), while internal rotation was 85° both actively and passively. Task-based assessment was available at the last clinical review for all 12 patients, including those who later died of disease: 11 of 12 could bring the hand to the mouth, nine of 12 could bring the hand to the head, and ten of 12 could reach the ipsilateral pocket.

### Complications, cosmetic outcome, and donor-site morbidity

The main reconstructive complication was pseudarthrosis, as described above. No major donor-site morbidity, ankle instability, gait disturbance, motor deficit, or deformity was observed. One patient reported donor-site pain, and one patient reported recurrent oedema during sports practice. Cosmetic dissatisfaction was reported by all living patients and was mainly related to loss of shoulder contour, visible asymmetry, and prominence of the reconstructed shoulder girdle, particularly in the coracoid region (Fig. 2). All living patients expressed interest in secondary cosmetic reconstruction, and one subsequently underwent secondary contour reconstruction using a silicone implant, with a satisfactory patient-reported cosmetic result. The remaining patients had not undergone secondary cosmetic surgery at the latest follow-up. No graft fracture, implant failure, deep infection, or vascular graft failure was observed.

## Discussion

This multicentre retrospective study showed that shoulder arthrodesis using a vascularized fibular graft can provide a stable and functionally useful limb after proximal humeral tumour resection involving the rotator cuff and functional deltoid mechanism. Among living patients, the mean MSTS score was 85%. Although true shoulder abduction was minimal, functional elevation was achieved mainly through scapulothoracic compensation, allowing most patients to perform essential tasks.

Reconstruction after proximal humeral tumour resection remains difficult because functional outcome depends on both skeletal reconstruction and the residual soft-tissue envelope. Several reconstructive strategies have been described, but they are not directly interchangeable [2, 14]. Instability, allograft fracture, and graft-host non-union remain important limitations of prosthetic, osteoarticular allograft, and allograft-prosthetic composite reconstruction, respectively [2, 14, 15]. The choice of reconstruction should therefore be guided by tumour extent, prognosis, functional demand, and preservation of the rotator cuff, deltoid, and axillary nerve.

Reverse shoulder arthroplasty has expanded reconstruction after proximal humeral tumour resection, particularly when the rotator cuff is deficient but the deltoid and axillary nerve remain functional. In selected patients, it can restore active elevation and may provide better motion than hemiarthroplasty or conventional endoprosthetic reconstruction [6, 7, 16, 17]. However, when the deltoid insertion, axillary nerve, capsule, or surrounding soft tissues are compromised, outcomes become less predictable, with instability, loosening, and revision surgery remaining important concerns [7–9]. Favourable results in selected patients should therefore not be directly extrapolated to patients in whom the rotator cuff and functional deltoid mechanism cannot be preserved.

In the present series, resection consistently compromised the rotator cuff and functional deltoid mechanism. The aim of reconstruction was therefore not restoration of glenohumeral motion, but creation of a stable shoulder girdle that allowed useful positioning of the hand. This distinction is important when interpreting function after arthrodesis. Mean true shoulder abduction was only 7°, whereas mean active scapulothoracic abduction was 75°. Functional movement was therefore generated mainly by scapulothoracic compensation rather than by glenohumeral motion. In this setting, task-based outcomes may be more clinically meaningful than isolated range-of-motion values, because the primary goal is independent use of the hand in space.

The functional results of this series are consistent with previous experience with shoulder arthrodesis after extensive proximal humeral tumour resection [4, 10, 18, 19]. In our study, the mean MSTS score was 25.5/30, with active flexion of 80° and active scapulothoracic abduction of 75°. These results compare favourably with published outcomes, although direct comparison is limited by differences in resection type, soft tissue sacrifice, age, tumour diagnosis, and follow-up [2, 4, 10, 14, 15, 18, 19].

Biological reconstruction with a vascularized fibular graft offers specific advantages in young patients and large oncological defects. The graft provides living autologous bone with potential for incorporation, hypertrophy, and remodelling under mechanical load. In upper-extremity tumour reconstruction, vascularized fibular grafting has been associated with high union rates and good functional outcomes, although reoperation and mechanical complications remain frequent [20–22]. Although final union was achieved in two-thirds of patients, union was often delayed and sometimes required revision surgery. This likely reflects the demanding biological and mechanical environment of scapulohumeral arthrodesis, including long segmental defects, chemotherapy exposure, graft-host size mismatch, and the need for incorporation at both proximal and distal interfaces.

Pseudarthrosis was the main reconstructive complication and occurred exclusively at the distal graft-host junction. This consistent anatomical distribution suggests that the distal junction may represent the most vulnerable part of the construct. Potential contributing factors include graft-host diameter mismatch, limited cortical contact, fixation stability, graft length, chemotherapy exposure, and rotational stress at the junction between the slender fibular graft and the larger humeral diaphysis. However, because of the small cohort and limited number of events, these factors could only be assessed descriptively, and no independent association with pseudarthrosis could be established. Fibular graft hypertrophy was observed in surviving patients, with a mean hypertrophy of 34%, whereas hypertrophy was more limited in patients who died early from disease. These findings support careful preparation of the distal docking site, optimisation of graft-host contact, stable fixation, and continued radiological surveillance until union is achieved.

Compared with prosthetic reconstruction, biological arthrodesis avoids prosthetic dislocation and loosening but introduces a different spectrum of complications, including delayed union, pseudarthrosis, graft fracture, donor-site symptoms, and cosmetic deformity. Cement spacers may be useful in patients with limited expected survival or poor soft-tissue function, although they do not provide biological incorporation or remodelling [3]. Osteoarticular allografts and allograft-prosthetic composites preserve bone stock but remain limited by fracture, degeneration, and graft-host non-union [2, 14, 15, 23]. Clavicula-pro-humero reconstruction represents another option in selected paediatric patients or when microsurgical reconstruction is unavailable, although mechanical complications and limb shortening may occur [24]. Vascularised fibular graft arthrodesis therefore occupies a specific reconstructive role when the rotator cuff and functional deltoid mechanism cannot be preserved. It provides biological reconstruction and a stable platform for scapulothoracic positioning, but at the cost of absent glenohumeral motion, prolonged time to union, pseudarthrosis risk, and cosmetic asymmetry.

Cosmetic dissatisfaction was reported by all living patients and was mainly related to loss of the normal shoulder contour, visible asymmetry, and prominence of the reconstructed shoulder girdle, particularly around the coracoid region. This finding is clinically important, especially in children, adolescents, and young adults, and should be discussed explicitly during preoperative counselling. Patients and families should understand that the principal aim of the procedure is to obtain a stable and functionally useful limb rather than to restore normal shoulder appearance or glenohumeral motion. Potential strategies to reduce cosmetic concerns include meticulous soft-tissue coverage at the index procedure, preservation of viable soft tissues whenever oncologically safe, and consideration of delayed secondary contour reconstruction after oncological stability has been established. One patient underwent secondary contour reconstruction using a silicone implant and reported satisfaction with the cosmetic result. However, as this was an isolated case, no conclusions can be drawn regarding the effectiveness or durability of this approach.

This study has several limitations. Its retrospective design and small sample size reflect the rarity of this reconstructive indication and limit the strength of statistical inference. The cohort size did not permit reliable identification of independent risk factors for pseudarthrosis or other complications, and the findings should therefore be interpreted primarily as descriptive. The absence of validated patient-reported outcome measures is an additional limitation, because the clinician-reported MSTS score does not fully capture patient-perceived disability, quality of life, satisfaction, or the psychosocial impact of cosmetic dissatisfaction. The absence of a control group also limits direct comparison with alternative reconstructive techniques. Differences in patient age, tumour histology, resection length, fixation construct, chemotherapy exposure, and duration of follow-up may affect the generalisability of the results, although most patients had osteosarcoma and all underwent reconstruction for the same anatomical and functional indication. Radiological assessment was based mainly on serial plain radiographs because CT was not systematically available. Functional assessment was performed in living patients at final follow-up, whereas task-based assessment was available for all patients at their last clinical evaluation. Future prospective studies should incorporate validated upper-limb and quality-of-life outcome measures.

## Conclusion

Despite its limitations, this multicentre study provides contemporary data on a rare biological reconstruction after extensive proximal humeral tumour resection. In carefully selected patients in whom the rotator cuff and functional deltoid mechanism cannot be preserved, shoulder arthrodesis using a vascularised fibular graft may provide a stable and functionally useful limb through scapulothoracic compensation. However, the procedure does not restore glenohumeral motion and is associated with prolonged time to union, a substantial risk of distal graft-host pseudarthrosis, and cosmetic dissatisfaction. It may therefore be considered a limb-salvage option in carefully selected patients with this specific anatomical and functional defect.

## Data Availability

No datasets were generated or analysed during the current study.
